# DWV-A Lethal to Honey Bees (*Apis mellifera*): A Colony Level Survey of DWV Variants (A, B, and C) in England, Wales, and 32 States across the US

**DOI:** 10.3390/v11050426

**Published:** 2019-05-09

**Authors:** Jessica L. Kevill, Flaviane S. de Souza, Christopher Sharples, Randy Oliver, Declan C. Schroeder, Stephen J. Martin

**Affiliations:** 1 School of Environment and Life Science, The University of Salford, Manchester M5 4WT, UK; jkevill@umn.edu (J.L.K.); flaviane.fss@gmail.com (F.S.d.S.); chrisdsharples@hotmail.co.uk (C.S.); 2 Veterinary Population Medicine, College of Veterinary Medicine, University of Minnesota, St Paul, MN 55108, USA; Dcschroe@umn.edu; 3 Center of Agrarin, Environmental and Biological Sciences, Federal University of Reconcavo of Bahia, Rui Barbosa 710, Cruz das Almas, Bahia 44380-000, Brazil; 4 ScientificBeekeeping.com, Grass Valley, CA 95945, USA; randysbees@gmail.com; 5 School of Biological Sciences, University of Reading, Reading RG6 6LA, UK

**Keywords:** deformed wing virus, *Varroa*, honey bees (*Apis mellifera*), overwinter colony loss, ABC assay

## Abstract

The strong association between *Varroa destructor*, deformed wing virus (DWV), and high overwintering colony losses (OCL) of honey bees is well established. Three DWV master variants (DWV-A, -B, and -C) have been described, and their role in colony mortality remains an open question. Therefore, the aim of this study is to investigate the seasonal prevalence, viral load, and changing distribution of the three DWV master variants within honey bee colonies from England, Wales, and 32 states across the United States. Here, we report that in 2016, DWV-B was prevalent (100%, *n* = 249) and dominant (95%) in England and Wales, compared to the US. (56%, *n* = 217 and 23%, respectively), where DWV-A was prevalent (83%, *n* = 217) and dominant (63%). DWV-C was regularly detected in low viral loads (<1 × 10^7^ genome equivalents per bee) and at lower prevalence (58% in England and Wales, *n* = 203, and 14% across the United States, *n* = 124) compared to DWV-A and -B. DWV-B prevalence and dominance in England and Wales coincided with low OCL (6%). Meanwhile, a 60% loss was reported by participating U.S. beekeepers. In the United States, DWV-A prevalence (89%, *n* = 18) and viral load were significantly (*p* = 0.002) higher (1 × 10 ^8^–1 × 10^11^) in colonies that died when compared to the surviving colonies (49% (*n* = 27), 1 × 10^6^–1 × 10^10^). DWV-B had low prevalence (56%, *n* = 18) in the colonies that died with viral loads of <1 × 10^10^. However, DWV-B was routinely detected in high viral loads (>1 × 10^10^) in surviving colonies from all sample locations, providing further supporting evidence of DWV-A exhibiting increased virulence over DWV-B at the colony level.

## 1. Introduction

Declines in both wild and domesticated pollinators are of growing concern [1]. Multiple stressors such as habitat loss, pesticides, and pests are implicated in the decline of honey bees [1]. One pest in particular, commonly referred to as simply “Varroa”, is now a major driver in the loss of colonies world-wide [1,2,3,4]. “Varroa” here actually refers to a particular species of Varroa mite, *Varroa destructor* [5]. *Varroa jacobsoni* was originally described in Java and confined to South Asia [6]. The *V. jacobsoni* mite is a parasite of *Apis cerana* (eastern cavity nesting bee), but in the late 1950s, jumped the species barrier to *A. mellifera* when colonies were kept alongside those of *A. cerana* [7]. Later, the *V. jacobsoni* mites infesting colonies of European honey bees were shown to belong to a new species of mite, *V. destructor* [5]. *Varroa destructor* (here on in referred to as Varroa) now has a global distribution, with the exception of Australia. The introduction of Varroa to European honey bees has transformed a relatively benign virus, deformed wing virus (DWV), to become one of the most widespread and destructive insect pathogens on the planet [2,3,4].

Normally, DWV persists in a variety of insect hosts at low prevalence, low viral load, and high strain diversity [2,8,9]. However, the introduction of new mite-meditated DWV transmission allowed for at least one of the three DWV master variants (A, B, or C) to be selected in honey bees [2,9]. Initially, only DWV-A was detected, and became closely associated with the collapse of colonies around the world [2,10,11]. DWV-B was first detected in Varroa in 2001 [12], and was believed to be a mite-borne virus. It was originally named Varroa Destructor virus-1 (VDV-1). Subsequent studies have shown VDV-1 to be a master variant of DWV [13,14], now called DWV-B, as it is commonly detected in honey bees [4]. The virulence of DWV-B in honey bees remains unresolved since Mordecai et al. [15] suggested that, at the colony level, DWV-B can protect the bees via superinfection exclusion (SIE), which prevents the more virulent DWV-A from prevailing within a colony. Meanwhile, other research has found DWV-B to be equally or more virulent than DWV-A when injected in high viral loads (>1 × 10^7^) to individual DWV-naïve young adult bees in cage experiments [16,17,18]. DWV-C was discovered in U.K. honey bee samples from 2007 [14], and linked in combination with DWV-A to the death of overwintering colonies in which the mite populations were controlled at low levels [19,20]. 

The northern hemisphere honey bee populations always suffer greater losses than populations in the southern hemisphere. This is largely due to the presence of naturally occurring mite-tolerant African or Africanised honey bees found throughout Africa [21], most of South America, and some states within Central America, whilst in Australia Varroa and DWV are absent [22]. In the northern hemisphere, the United States consistently experiences the greatest overwinter losses, ranging between 22 and 35% over the last decade [23], whilst over a similar period, U.K. overwinter colony losses have ranged between 10 and 30% [24]. Beekeepers within the United States suggest that colonies are lost due to a number of factors, such as queen failure, starvation, poor weather, Varroa infestation, and pesticides [25,26,27,28,29,30,31,32]. These differences, along with changing perceptions of the beekeeper, create difficulties in assessing the true causes of colony losses, particularly during the overwinter period when, due to environmental conditions, it is rare to inspect colonies. The European scientific community commonly reports pathogens, especially RNA viruses, in the presence of Varroa to be a major factor in overwintering colony losses (OCL) [11,19,33,34,35,36,37], with studies in the United States also providing evidence that pathogens are linked to weakened colonies and colony losses [2,38,39,40]. 

There are few landscape scale comparative studies that directly focus on DWV seasonal prevalence and their impact at colony level. To directly address this research gap, we studied the distribution, prevalence, and viral load of the three DWV variants (DWV-A, -B, and -C) at colony level in England, Wales, and 32 states across the United States, using the ABC assay [20]. This study sheds new light on the dynamic nature of the DWV master variants. The mortality of the study colonies was also recorded to help further understand the impact of DWV master variants at the colony level.

## 2. Materials and Methods 

### 2.1. Sample Collection 

**Figure 1:**
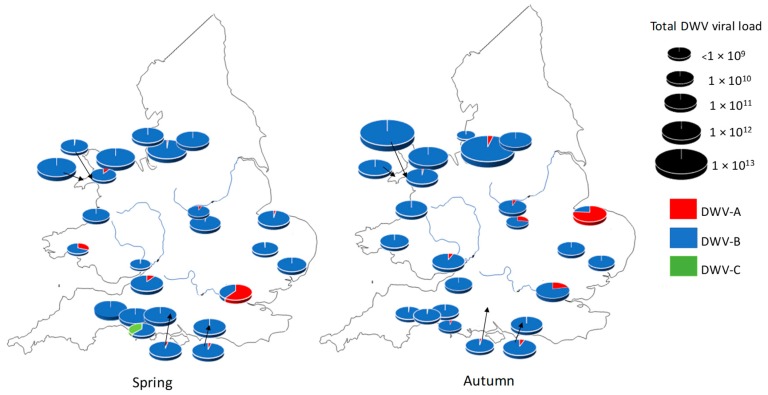
A map of England and Wales showing the average DWV load and variant per corresponding apiary in the spring and autumn of 2016. Size of pie chart is relative to total DWV load per apiary.

A total of 35 English and Welsh apiaries were randomly selected from beekeepers belonging to British beekeepers’ regional associations, who independently volunteered samples. The apiaries were sedentary and located over a broad geographical range (Figure 1). The first samples were collected on the initial inspection in April 2016 (spring) and a second sample from the same colonies between mid-August and mid-September 2016 (autumn). A total of 29 and 30 apiary samples were obtained in spring and autumn respectively, resulting in 24 paired apiaries. In total, 249 samples were received from English and Welsh colonies. Similarly, 78 beekeepers across the United States were contacted by Randy Oliver and asked to sample three colonies per apiary, prior to the overwinter period of November 2016. Again, 50 adult honey bee samples were collected from the brood area of 217 honey bee colonies. Samples were received from 32 states across the United States. Samples were collected mainly from the apiaries of hobbyist beekeepers (*n* = 64), whilst some samples were provided by commercial apiaries (*n* = 14). 

A health questionnaire was sent to all the beekeepers who provided samples and was completed in the spring of 2017. The health questionnaire asked for the number of colonies lost during the overwintering period and the details surrounding any losses. A total of 25 English and Welsh, and 35 U.S. beekeepers returned a completed questionnaire. 

### 2.2. Sample Processing 

Each sample was manually checked for Varroa mites and any visual deformities to the bee, and both were removed when found. Pools of 20 asymptomatic whole worker bees were homogenised in liquid nitrogen, using a mortar and pestle for the English and Welsh honey bee samples; however, to speed up sample processing, due to the large number of U.S. samples, a pool of ten bee heads was used for analysis. Preliminary analysis confirmed that using the heads, or whole bodies, provided comparable data (Supplementary Figure S1) and is supported by previous studies that have used bee heads for DWV [41,42,43], acute bee paralysis virus [44], and Kakugo virus [45]. 

The homogenate was stored at −80 °C until RNA extraction was conducted. RNA was always extracted from 30 mg of honey bee tissue from the homogenised pool of bees, using an RNeasy Mini prep kit (Qiagen). RNA was then quantified on Nanodrop 2000 (Thermo Fisher Scientific) and standardised to 50 ng/µl per sample. 

### 2.3. RT-qPCR

RT-qPCR was performed on the 1 μL total RNA (50 ng/μL) extracted from a pool of 20 adult honey bees (England and Wales), or 10 heads (United States), using the ABC assay method [20]. 

Samples were analysed in triplicate and any samples that had a 3 Ct value deviation between triplicates were removed from the analysis, as 3 Ct values are the equivalent to a ten-fold increase or decrease in viral load. A total of 249 colonies located in England and Wales and 217 colonies in the United States were screened for each DWV master variant. 

### 2.4. Analysis of the Results 

The viral load is expressed as genome equivalents per honey bee, using the following equations [20]:

Copy number RNA = (Concentration RNA (ng/µL) × 6.022 × 10^23^)/(Fragment length base pairs × 109 × 325) (1)

Genome equivalents = (average copy number) × (RNA dilution factor) × (elution volume of RNA) × (proportion of bee material)(2)

The viral load of DWV variants was converted into percentages across the three colonies and averaged for the apiary. Each apiary is represented by a pie chart, the size of the pie chart reflecting the total DWV viral load, which was plotted onto the relevant map. The exact geographical location is not given to protect the beekeepers’ identity. Statistical analysis was conducted using non-parametric tests, as the viral load data were not normally distributed. A Kruskal–Wallis test was used to compare the viral load, per DWV variant. Comparisons between spring and autumn were made for the English and Welsh colonies, and between surviving colonies, or colonies that died during the winter of 2016/17, for England, Wales, and the United States. Post hoc analyses were conducted using a Dunn’s test of multiple pairwise comparisons [46]. The significance threshold was set at *p* < 0.05, and when multiple comparisons were made, the significance level was adjusted using a Bonferroni correction (significance threshold/number of comparisons). Significant results were those that returned a *p*-value of <0.025. 

## 3. Results

### 3.1. DWV Variant Prevalence and Viral Load Between Spring and Autumn in Apiaries in England and Wales 

The apiary level analysis reveals that between spring (*n* = 29) and autumn (*n* = 30), all apiaries tested positive for DWV-B and 99% tested positive for DWV-A. On the other hand, DWV-C was detected in 93% of apiaries for both time points. Although the prevalence appeared to be very similar, the apiary average DWV viral load in colonies differed for each variant, following the pattern of DWV-B > DWV-A > DWV-C for both spring and autumn (Table 1). 

Regardless of season, DWV-B was detected in high viral loads and dominated apiaries. DWV-A was dominant in only two apiaries in spring and two different apiaries in summer (Figure 1). DWV-C was present at less than 0.7% of the total DWV viral load (Figure 1), except in a single apiary in spring, where 38% of the viral load was represented by DWV-C. Here, 50% of colonies screened in this apiary tested positive for DWV-C; however, this was not maintained, since in autumn, DWV-B became dominant within the apiary (Figure 1).

### 3.2. English and Welsh Colony Level DWV Analysis 

**Figure 2:**
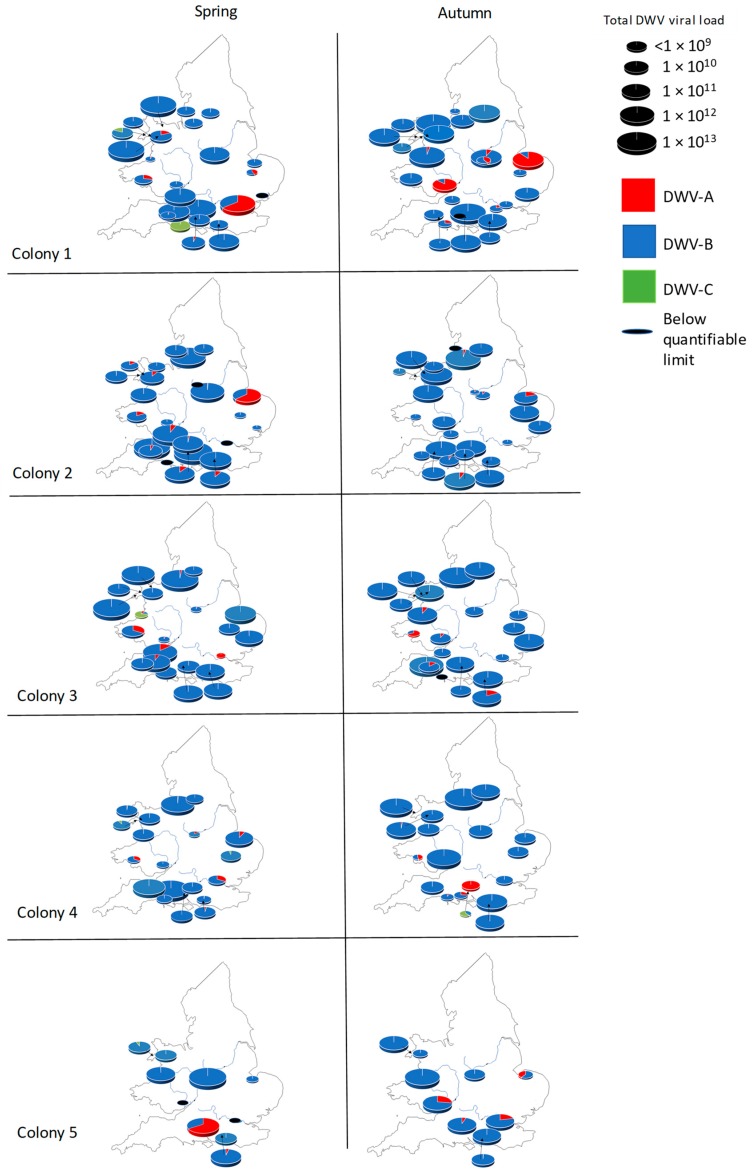
DWV variant prevalence and abundance in corresponding English and Welsh colonies, per season. Pie charts are representative of each colony sampled per apiary. The same colonies were sampled in spring and autumn. The size of the pie chart is relative to total DWV load per colony.

Colony level analysis of the RT-qPCR data for the English and Welsh samples reveals that across both sampling points (spring and autumn), every colony (*n* = 249) tested positive for DWV-B and 99% tested positive for DWV-A (Figure 2). Only 16% of colonies had a >10% proportional load of DWV-A, whereas DWV-B was detected at >50% proportional load in 88% of colonies. DWV-C was detected in 58% of colonies, although it was only quantifiable in 16% of colonies. DWV-B remained both prevalent and dominant from spring to autumn, with proportional viral loads exceeding 50% in 85% of colonies in spring, and 89% in autumn (Figure 2). DWV-A was dominant in 3% of non-corresponding colonies in both spring and autumn. Meanwhile, 0.75% of colonies in spring and 0.4% in autumn had DWV-C (*n* = 203) as the main DWV variant (Figure 2). 

**Figure 3:**
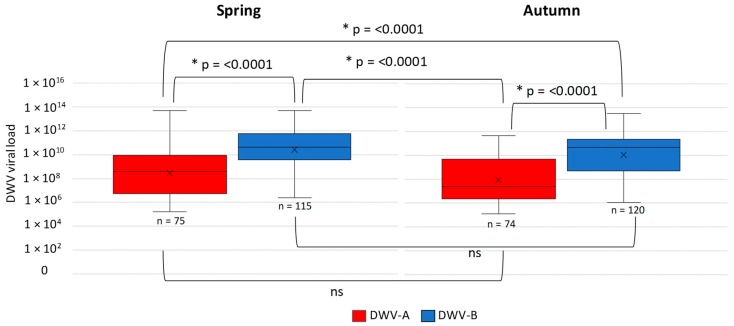
Colony level DWV-A (red) and -B (blue) viral load per season in RT-qPCR quantifiable colonies from England and Wales. Pairwise comparisons [46] are shown alongside non-significant (ns) and significant (*) results.

DWV-C rarely dominated a colony and quantifiable colonies were rare in the data. Therefore, a comparison of DWV-A and -B viral load between spring and autumn was conducted using a Kruskal–Wallis test. The result revealed at least one significant difference between groups (*n* = 384, H = 93.01, df = 3, *p* = <0.00001). Further post hoc analysis shows that DWV-A and -B viral loads between spring and autumn are consistent; however, when comparing the viral loads of DWV-A with DWV-B, significant differences occurred, as DWV-B viral loads are consistently higher than those of DWV-A between seasons (Figure 3).

### 3.3. Apiary Level Analysis of Pre-Winter United States Honey Bee Colonies

**Figure 4:**
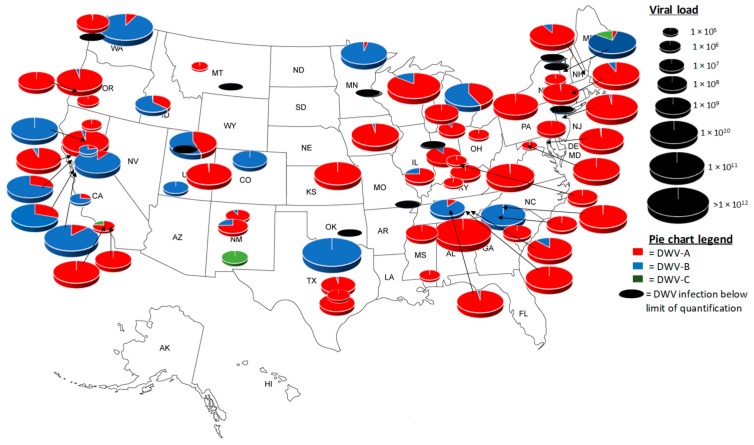
A map of the United States showing the average DWV load and variant per apiary in pre-wintering honey bees, 2016. Size of pie chart is relative to total DWV load per apiary.

Analysis of the colonies tested within the 78 apiaries prior to the overwintering period reveals that DWV is widespread (Figure 4). DWV-A was dominant in 63% of apiaries, whilst only 23% were DWV-B dominated. DWV-C was rare; therefore, we tested 124 colonies from the 78 apiaries sampled. Of these, only 1% were DWV-C positive. The remaining 13% of apiaries were positive below the limit of quantification for any DWV variant. The average viral loads between DWV-A and -B were similar, whilst DWV-C viral loads were low in comparison to those of DWV-A and -B (Table 2). Prevalence of DWV variants at the apiary and colony level reveals a pattern of DWV-A > DWV-B > DWV-C (Figure 4); however, the DWV viral loads revealed a pattern of DWV-A = DWV-B > DWV-C (Table 2).

### 3.4. Overwinter Colony Mortality 

**Figure 5:**
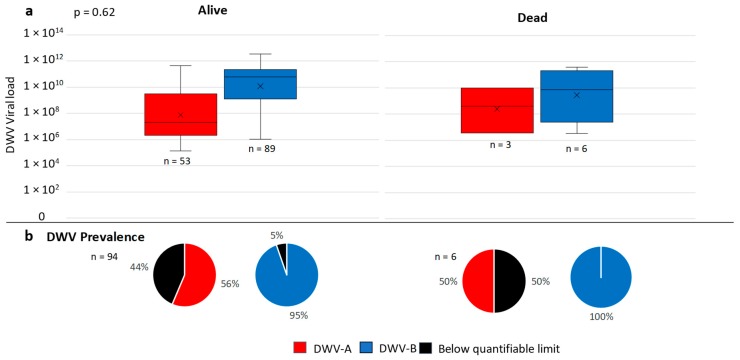
Box plots of viral load in DWV-A and -B RT-qPCR quantifiable English and Welsh colonies that survived the overwinter period and those that died (**a**). Pie charts show the percentage of DWV quantifiable colonies and those that tested positive below the limit of quantification (**b**).

The returned health questionnaires for the English and Welsh colonies revealed that mortality was recorded in 9 out of 100 (9%) colonies for both sampling points. Three colonies died in spring; one from robbing and two of no apparent cause. Six colonies died during the overwinter period (Figure 5). A comparison of total DWV loads in the autumn samples was made between known surviving (*n* = 94) colonies and those that died (*n* = 6), no statistical difference was found between total DWV load in the dead or surviving colonies (Kruskal–Wallis *n* = 97, H = 0.25, df = 1, *p* = 0.62). Furthermore, viral loads in dead colonies did not exceed those of surviving colonies, and prevalence was similar; therefore, these deaths could not be attributed to DWV infection alone (Figure 5). The questionnaire respondents provided details surrounding cause of death for the six colonies and this was considered to be the effect of bad weather, caused by Storm Doris. 

**Figure 6:**
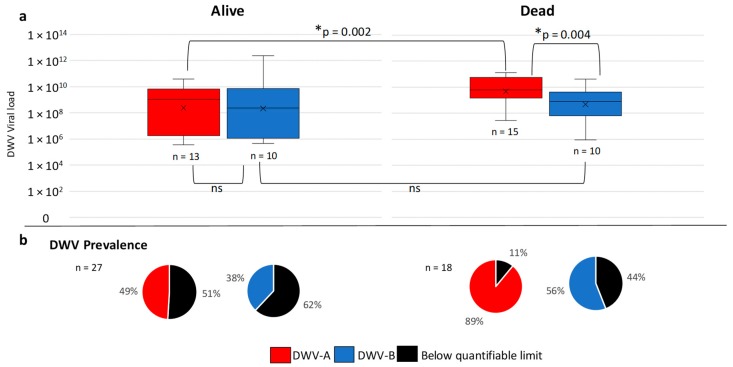
Box plots of viral load for DWV-A and -B in RT-qPCR quantifiable U.S. colonies that survived the overwinter period and those that died (**a**). Significant (*) and non-significant comparisons (ns) are shown. Pie charts show the percentage of DWV quantifiable colonies and those that tested positive below the limit of quantification (**b**).

Data on colony mortality were obtained from 45 U.S. colonies, of which 27 died and 18 survived the overwintering period. Statistical analysis revealed at least one significant difference between the groups (alive or dead) (Kruskal–Wallis H = 15.2, df = 3, *p* = 0.0016). Additional post hoc analysis using a Dunn’s test [46] revealed that DWV-A viral load was significantly (*p* = 0.002) higher in the colonies that died (1 × 10^8^–1 × 10^11^) (Figure 5) than in those that survived (1 × 10^6^–1 × 10^10^) (Figure 5), whilst DWV-B loads were significantly (*p* = 0.004) lower (1 × 10^6^–1 × 10^10^) (Figure 6) than DWV-A in the colonies that died (Figure 6). There were no significant differences between DWV-A and DWV-B viral loads in the surviving colonies and DWV-A and -B prevalence was reduced (49% and 38%, respectively) (Figure 6). However, an increase in the prevalence of DWV-A and -B quantifiable colonies (89% and 56%, respectively) occurred in the colonies that died during the overwinter period (Figure 6).

## 4. Discussion

The DWV-B variant was widespread and dominant in English and Welsh honey bee colonies, a situation that was maintained throughout the beekeeping season (spring to autumn) of 2016. DWV-A was widespread and dominant in the United States, and was also reported as the prevalent DWV master variant in 75 U.S. colonies sampled in 2010, when only 2.7% of colonies tested positive for DWV-B [47]. DWV-B is increasing in prevalence as 23% of U.S. apiaries were dominated in our data set, and 66% tested positive in the Ryabov et al. study [47]; however, DWV-A remained the prevalent DWV variant. The presence of DWV-A in English and Welsh colonies, and its dominance in samples collected in 2007 in the absence of DWV-B [19,20], shows that DWV master variants have undergone recent selection events, allowing DWV-B to become the prevalent and most common DWV variant in England and Wales, a situation that is predicted to occur in the United States.

The English and Welsh honey bee colonies sampled in spring survived the winter of 2015/2016, and entered the 2016 beekeeping season with DWV-B viral loads in the range of 1 × 10^6^–1 × 10^14^. The results reveal that DWV-B loads were significantly higher (*p* ≤ 0.00001) than DWV-A at both spring and autumn sampling in England and Wales. A similar result was also witnessed in two German apiaries. Again, honey bee colonies survived the winter period with DWV-B infections; however, the loss of overwinter worker bees was reported [48]. Our data show that DWV loads in English and Welsh colonies remained consistent throughout the season, with a large number (96%) of colonies surviving the winter period of 2016/2017, where 63% of these survivor colonies had DWV-B viral loads >1 × 10^10^ genome equivalents per bee (Supplementary Table S1). Furthermore, overwinter colony losses (OCL) reported by The British Bee Keepers Association (BBKA) for our sample year were the second lowest in a decade at 13% [24]. The English and Welsh OCL reported in our survey cannot be attributed to DWV. Instead bad weather caused by Storm Doris was the most likely cause of death, as reported by the participating beekeepers. The effect of DWV-B at colony level cannot be considered catastrophic at this time. No significant differences were witnessed for DWV-B viral load between those colonies that died or survived in the English and Welsh data, as well as the U.S. data. DWV-B was also the dominant variant in a long-term surviving Varroa-resistant population of honey bees in the United Kingdom, leading to the suggestion that this variant excludes more lethal DWV-A via superinfection exclusion (SIE) [15]. SIE occurs when a virus has the ability to successfully outcompete and exclude other closely related variants, and has been well documented in hepatitis C [49]. The results presented here suggest the potential for DWV-B and SIE to be a phenomenon beyond the confines of a single apiary in Swindon, England. Other apiaries in England and Wales, and now the United States, may also be experiencing this phenomenon of protection against the more virulent DWV-A. 

The U.S. honey bee population suffers severe overwinter losses in the range of 22–35% periodically [23], and the participating beekeepers in our mortality survey confirmed that 60% of sampled colonies were lost in the winter of 2016/2017. Our data show a significant difference in DWV-A viral load between colonies that survived and those that died (*p* = 0.002). Here, we provide further evidence that DWV-A is lethal to colonies, as DWV-B viral load was significantly lower than that of DWV-A in the colonies that died (*p* = 0.004). In addition, DWV-A and -B prevalence was also reduced in the colonies that survived (49% and 38%, respectively), whilst 89% of the dead colonies had dominant DWV-A infections. In 2009, Highfield et al. [19] clearly demonstrated that high DWV loads were present in colonies that failed to survive the overwinter period and, in 2017, these samples were rescreened using the ABC assay [20]. The ABC assay data provided corroboration that DWV-A and -C co-infections in high viral loads were behind OCL. DWV-B was found to be low (<1 × 10^7^) in colonies that died from DWV-A and -C infection in 2007 [20]. This was also the year in which colony losses were the highest in the United Kingdom at 30% [23]. Based on the evidence reported here, and also by Mordecai et al. [15], it would be expected that an increase in DWV-B prevalence and dominance, accompanied by a decrease in DWV-A, could reduce the incidence of OCL in the US. 

While the results presented in this study show a high number of colonies surviving with DWV-B dominated infections, others have shown DWV-B to be lethal to individual bees [16,17,18,48]. High DWV-B (>1 × 10^10^) viral loads were shown to kill inoculated caged bees and pupae [16,18]. These studies mimic Varroa-mediated transmission of DWV, as bees were directly injected with DWV variants and clearly demonstrate that Varroa feeding activities as the main transmission route are lethal. At the colony level, several other viral transmission routes exist [50,51,52,53], and bees within a colony are of various ages; therefore, DWV-B may not impact the colony as much as it will an experimentally injected individual. In colonies where Varroa mites are controlled, DWV viral loads have been shown to be reduced after treatment, but then increase to subclinical levels, demonstrating that non-Varroa transmission of DWV is also important for maintaining DWV infections within a colony [54]. It is hypothesised that DWV-B viral loads must exceed the threshold of host tolerance in all adult bees to kill a colony or that co-infection with a more lethal variant must occur. Further studies of this nature are required to ascertain when a DWV-B infection becomes lethal to honey bee colonies. The U.S. data set provides the perfect opportunity to further assess the role of DWV-B at colony level, as it is predicted that DWV-B will eventually become the dominant DWV variant in the United States.

Overall, we have shown that DWV-B was prevalent in England and Wales in 2016, and that this coincided with a low number of colony losses. Furthermore, viral loads were maintained between seasons. DWV-A was detected in significant viral loads in colonies that died in the United States and is still considered lethal, as demonstrated in previous studies [2,19,20]. 

## Figures and Tables

**Table 1:** Mean apiary level deformed wing virus (DWV) viral load and standard error (SE), per season and variant in English and Welsh colonies.

	Spring	SE Spring	Autumn	SE Autumn
**DWV-A**	3.89 × 10^11^	7.48 × 10^10^	2.46 × 10^10^	4.46 × 10^9^
**DWV-B**	1.48 × 10^12^	2.26 × 10^11^	1.14 × 10^12^	2.01 × 10^11^
**DWV-C**	2.18 × 10^9^	5.83 × 10^8^	1.80 × 10^9^	5.21 × 10^8^

**Table 2:** Mean apiary level DWV viral load and standard error (SE), per variant in U.S. colonies.

	Average Viral Load	SE
**DWV-A**	1.74 × 10^10^	2.16 × 10^9^
**DWV-B**	7.12 × 10^10^	1.05 × 10^10^
**DWV-C**	1.99 × 10^8^	5.53 × 10^7^

## Supplementary Materials

The following are available online at https://www.mdpi.com/1999-4915/11/5/426/s1, Figure S1: RT-qPCR data of DWV viral loads detected in the heads and bodies of individual adult honey bees. Table S1: The DWV master variants and total viral load in colonies that survived or died. Samples were collected in late summer, 2016, from England and Wales. BL = below the quantifiable limit of detection.
