# Supplementary material for: DWV-A Lethal to Honey Bees (Apis mellifera): A Colony Level Survey of DWV Variants (A, B, and C) in England, Wales, and 32 States across the US
Source: Viruses. 2019 May 9;11(5):426. doi: 10.3390/v11050426 (PMC6563202; doi:10.3390/v11050426)
Supplement: Supplementary file 1 [file viruses-11-00426-s001.pdf]

Supplementary Data

Figure S1. RT-qPCR data of DWV viral loads detected in the heads and bodies of individual adult honey bees.

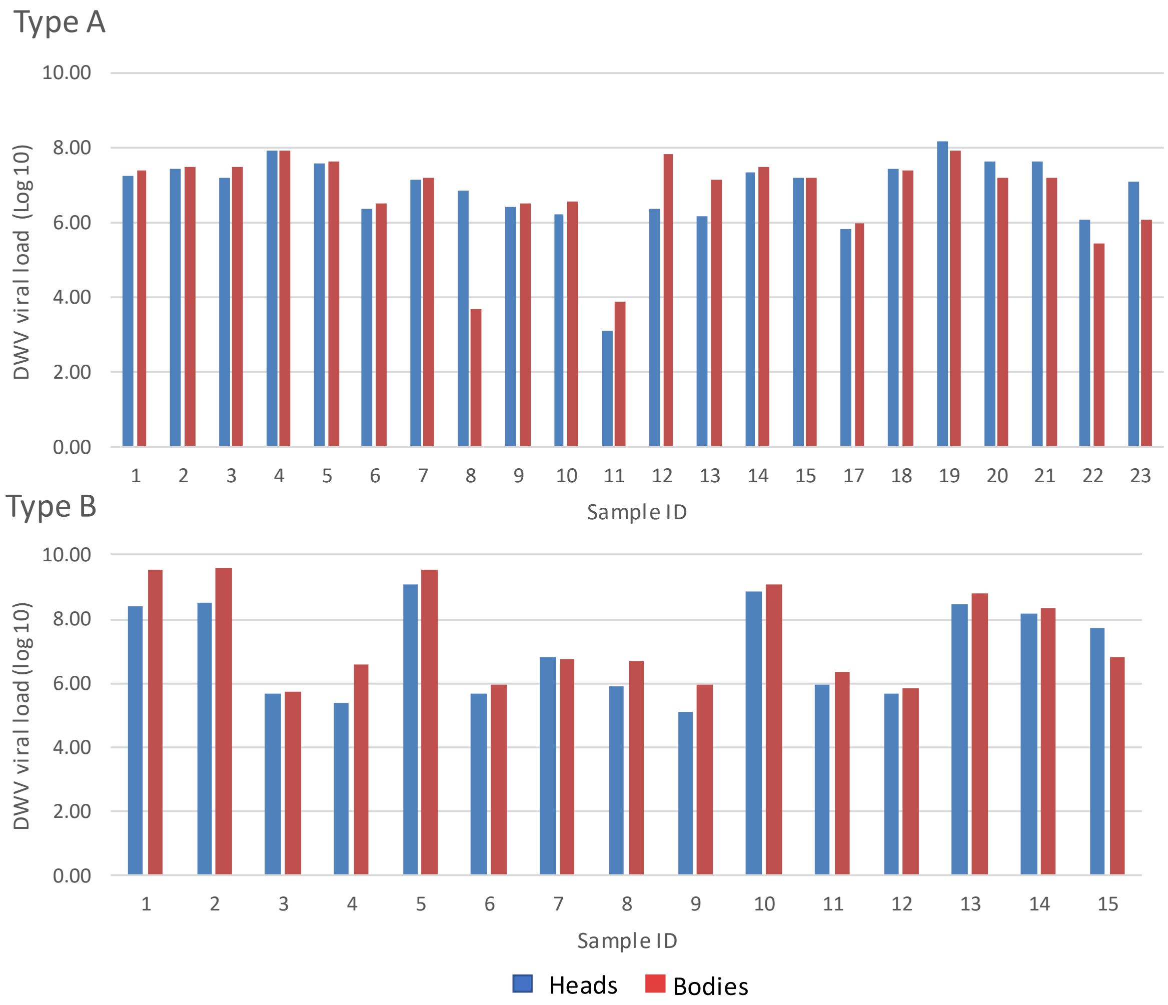

Table S1. The DWV master variants and total viral load in colonies that survived or died. Samples were collected in late summer 2016 from England and Wales. BL = below the quantifiable limit of detection.

Survivorship	ID	A	B	C	Total
Dead	LH1	3.91E+08	1.60E+09	BL	1.99E+09
Dead	AB3.2	3.69E+06	1.70E+11	NEG	1.70E+11
Dead	AB4.2	BL	3.31E+10	BL	3.31E+10
Dead	NLBK1#2	BL	4.84E+07	NA	4.84E+07
Dead	TREE2#2	1.01E+10	3.33E+06	6.65E+06	1.01E+10
Dead	TREE3#2	BL	3.74E+11	1.36E+07	3.74E+11
Alive	LH2	1.76E+08	BL	BL	1.76E+08
Alive	LH5	1.41E+10	1.97E+11	BL	2.12E+11
Alive	LH6	2.81E+08	2.66E+10	BL	2.68E+10
Alive	LH8	1.03E+09	1.03E+12	BL	1.04E+12
Alive	BTG	BL	1.14E+10	BL	1.14E+10
Alive	NE2	1.39E+07	2.00E+07	NA	3.39E+07
Alive	NE1	1.64E+06	2.09E+07	NEG	2.26E+07
Alive	TT5	2.52E+06	9.62E+06	NEG	1.21E+07
Alive	TT52	9.75E+08	6.85E+11	BL	6.86E+11
Alive	TT7	1.43E+09	4.88E+11	NA	4.89E+11
Alive	TT9	1.99E+11	5.05E+07	NA	1.99E+11
Alive	TT6	3.35E+08	4.06E+07	NEG	3.75E+08
Alive	IP5.2	BL	6.53E+10	NEG	6.53E+10
Alive	IP7.2	BL	2.45E+10	BL	2.45E+10
Alive	IP4.2	BL	1.03E+11	NEG	1.03E+11
Alive	SW9	6.71E+05	8.89E+09	BL	8.89E+09
Alive	SW3.1	2.58E+06	6.12E+10	BL	6.12E+10
Alive	SW2.1	1.96E+09	1.33E+10	BL	1.53E+10
Alive	SW5	BL	5.61E+10	BL	5.61E+10
Alive	CB88.2	BL	9.06E+07	BL	9.06E+07
Alive	CB3.2	BL	1.32E+11	NA	1.32E+11
Alive	CB12.2	BL	2.12E+10	NEG	2.12E+10
Alive	CB8.2	BL	2.09E+09	BL	2.09E+09
Alive	DFBKA19#2	1.29E+10	1.80E+09	NEG	1.47E+10
Alive	DFBK8.2	2.40E+06	9.12E+10	NA	9.12E+10
Alive	DFBK9.2	4.77E+09	6.50E+10	BL	6.97E+10
Alive	DFBK7.2	2.69E+09	2.04E+12	NA	2.04E+12
Alive	DFBK6.2	1.44E+11	3.76E+11	BL	5.20E+11
Alive	AB2.2	1.07E+11	3.52E+12	BL	3.62E+12
Alive	AB5.2	BL	3.15E+10	1.16E+08	3.16E+10
Alive	163.2	BL	2.84E+10	BL	2.84E+10
Alive	161.2	1.30E+09	8.34E+11	7.52E+06	8.36E+11

Alive	153.2	BL	2.04E+12	NEG	2.04E+12
Alive	WD2.2	1.40E+07	4.26E+07	NEG	5.67E+07
Alive	WDL2	6.11E+07	1.47E+09	3.23E+06	1.54E+09
Alive	WD12.2	BL	BL	BL	BL
Alive	WD7.2	2.01E+06	3.31E+07	NA	3.51E+07
Alive	98.2	BL	1.79E+12	NA	1.79E+12
Alive	A1.2	BL	1.92E+11	NA	1.92E+11
Alive	R1.2	2.20E+07	6.10E+11	BL	6.10E+11
Alive	20.2	9.08E+09	BL	BL	9.08E+09
Alive	66.2	1.17E+10	2.14E+11	BL	2.26E+11
Alive	fH1	BL	BL	BL	BL
Alive	SKH1#2	8.02E+06	4.09E+09	NEG	4.10E+09
Alive	NLBK6#2	BL	7.40E+06	NA	7.40E+06
Alive	NLBK8#2	5.42E+05	2.19E+08	NA	2.19E+08
Alive	NLBK9#2	1.35E+05	2.57E+09	BL	2.57E+09
Alive	NLBK10#2	1.18E+11	4.32E+11	NA	5.50E+11
Alive	BP1#2	BL	1.08E+06	BL	1.08E+06
Alive	BP2#2	BL	BL	BL	BL
Alive	WS4.2	2.63E+06	4.85E+11	1.49E+09	4.87E+11
Alive	WS9.2	BL	6.87E+09	BL	6.87E+09
Alive	WS8.2	1.09E+06	2.14E+11	NEG	2.14E+11
Alive	WS6.2	BL	1.87E+11	NEG	1.87E+11
Alive	WS1.2	2.05E+07	1.79E+11	NA	1.79E+11
Alive	ER1.2	BL	1.85E+11	BL	1.85E+11
Alive	ER2.2	BL	5.58E+10	BL	5.58E+10
Alive	ER8.2	1.29E+06	2.06E+11	2.70E+08	2.06E+11
Alive	ER4.2	BL	6.60E+11	BL	6.60E+11
Alive	ECH10.2	BL	7.63E+10	BL	7.63E+10
Alive	ECH2.2	BL	2.16E+06	NEG	2.16E+06
Alive	ECH4.2	2.71E+07	1.28E+07	BL	4.00E+07
Alive	ECH5.2	BL	8.80E+06	BL	8.80E+06
Alive	ECH7.2	1.84E+06	2.03E+06	NA	3.87E+06
Alive	W1Y#2	4.45E+11	5.06E+10	NEG	4.96E+11
Alive	W4A#2	5.35E+09	2.20E+10	BL	2.73E+10
Alive	W4#2	2.15E+06	1.06E+09	NA	1.06E+09
Alive	W3#2	1.66E+07	6.72E+10	BL	6.72E+10
Alive	WN2#2	2.89E+07	1.95E+07	NEG	4.84E+07
Alive	C6	4.92E+06	3.02E+12	1.03E+09	3.02E+12
Alive	C8	BL	7.42E+09	BL	7.42E+09
Alive	C2	2.13E+06	5.49E+06	NA	7.62E+06
Alive	FEDW1#2	3.73E+05	4.16E+10	BL	4.16E+10
Alive	FEDW4#2	BL	3.92E+11	5.50E+07	3.92E+11
Alive	fEDW2#2	9.13E+05	7.28E+10	BL	7.28E+10
Alive	fEDW3#2	4.00E+09	2.68E+11	BL	2.72E+11

Alive	fEDW5#2	BL	1.19E+08	BL	1.19E+08
Alive	TREE1#2	1.15E+07	1.00E+11	NA	1.00E+11
Alive	TREE5#2	BL	1.43E+10	BL	1.43E+10
Alive	TREE4#2	BL	5.99E+10	5.80E+06	5.99E+10
Alive	WILLOW2	2.68E+06	1.76E+11	NA	1.76E+11
Alive	WILD2	1.08E+10	1.79E+11	BL	1.90E+11
Alive	garage 2	BL	9.46E+10	BL	9.46E+10
Alive	TRUNK2	BL	1.10E+07	BL	1.10E+07
Alive	ASTER2	BL	1.34E+07	2.03E+07	3.37E+07
Alive	W10H2	BL	2.73E+09	1.31E+07	2.74E+09
Alive	W14H2	BL	2.15E+11	BL	2.15E+11
Alive	W5H2	BL	2.45E+11	BL	2.45E+11
Alive	W12H.2	6.20E+06	2.79E+11	6.61E+07	2.79E+11
Alive	LB3#2	2.20E+06	2.24E+11	2.31E+07	2.24E+11
Alive	LB5#2	BL	4.91E+06	BL	4.91E+06
Alive	LB2.2	2.22E+05	2.32E+11	9.44E+07	2.32E+11
Alive	LB1#2	BL	3.53E+12	1.63E+08	3.53E+12
Alive	LB4#2	BL	3.27E+11	6.91E+07	3.27E+11
